# “White Cord Syndrome”: A Reperfusion Injury Following Laminectomy and Spinal Decompression Surgery—A Case Report and Literature Review

**DOI:** 10.1155/carm/5539165

**Published:** 2026-01-09

**Authors:** Iran Chanideh, Amirmohammad Khodaei, Masoud Ghadiri, Zeinab Shakibaee Fard, Mohammad Mehdi Falahi Tabar, Arian Yavari, Sepehr Niktash

**Affiliations:** ^1^ Clinical Research Development Center, Taleghani Hospital, Kermanshah University of Medical Sciences, Kermanshah, Iran, kums.ac.ir; ^2^ Student Research Committee, School of Medicine, Kermanshah University of Medical Sciences, Kermanshah, Iran, kums.ac.ir

**Keywords:** decompression, neurosurgery, reperfusion injury, white cord syndrome

## Abstract

White cord syndrome (WCS) is a rare but serious postoperative complication characterized by new neurological deficits and hyperintense signal changes on T2‐weighted magnetic resonance imaging (MRI) following spinal decompression surgery. Since it was first described by Chin et al. in 2013, WCS has been attributed to reperfusion injury resulting from sudden restoration of blood flow to chronically ischemic spinal cord tissue. Oxidative stress, microvascular thrombosis, and impaired autoregulation have been proposed as contributing factors. We report the case of a 61‐year‐old Iranian man with a history of cervical canal stenosis who developed quadriparesis and paresthesia following posterior decompression surgery. Postoperative MRI revealed hyperintense signals consistent with WCS. High‐dose methylprednisolone was administered immediately, leading to partial neurological recovery during hospitalization. This case highlights the importance of early recognition and aggressive management of WCS to improve functional outcomes. Raising awareness of this syndrome among spine surgeons is essential for timely diagnosis and management.

## 1. Introduction

White cord syndrome (WCS) is a rare postoperative complication characterized by acute neurological deterioration and hyperintense T2‐weighted magnetic resonance imaging (MRI) signals after spinal decompression in patients with chronic cervical canal stenosis [1]. The underlying mechanism is believed to involve ischemia–reperfusion injury, which occurs in the absence of direct intraoperative trauma [2]. Additional contributing factors may include occlusion of small spinal arteries, microthrombi formation, oxidative stress, and lipid peroxidation of neuronal membranes [3].

The term “WCS” was first introduced by Chin et al. in 2013, referring to the postoperative appearance of bright T2 hyperintensities within the spinal cord, which may be accompanied by severe neurological deficits [4, 5]. Since that initial report, an increasing number of cases have been documented, possibly due to greater awareness of the condition among clinicians.

This article presents a new case of WCS following posterior cervical decompression and provides a review of 20 previously published cases from 2013 to 2024 (Table 1), aiming to further enhance understanding of this uncommon but clinically significant condition.

**Table 1:** Previous cases reported regarding white cord syndrome.

	Year	Age/sex/comorbidity	Preoperative diagnosis (initial diagnosis)	Management (initial surgery)	IOM	Neurology after surgery	Onset of deficit	Management of WCS	Final clinical outcome
Chin et al. [5]	2013	59‐M	Large C5‐6 herniated disc	C4‐5 and C5‐6 (ACDF)	Yes. Diminished MEP signals following placement of the peek cage at C5‐6	Postoperative C6 incomplete tetraplegia with diminished sensory and motor function (ASIA C)	Immediately postsurgery	Immediate steroid treatment (hydrocortisone) and ongoing dexamethasone	At 16‐month follow‐up, the patient showed resistant weakness especially in the left side requiring assistance (ASIA D)

Giammalva et al. [6]	2016	64‐M	Severe cervical stenosis with voluminous C3‐C4 and C5‐C6 disc herniations	ACDF	Yes, sudden decrease in somatosensory and motor evoked potentials during the closure time	Severe tetraparesis, complete paraplegia, severe motor weakness, diffuse spastic hypertonia (ASIA A)	Immediate postoperative	HDS	Partial recovery (ASIA C)

Vinodh et al. [4]	2018	51‐F	Cervical extramedullary metastatic ductal carcinoma	Palliative cervical decompression and fusion, C2 to C5 laminectomy	No	Quadriplegia, loss of sensation to pain, temperature, touch, and proprioception (ASIA A)	Immediate postoperative period	HDS	No neurological improvement; remained quadriplegic (ASIA A)

Papaioannnou et al. [7]	2019	79‐M Hypertension Atrial fibrillation	Cervical spondylotic myelopathy (CSM) with posterior stenosis from C4 to C6	Posterior decompression from C3 to 6 and posterior fusion with lateral mass screws from C2 to C7	Yes, no intraoperative neurologic worsening	Inability to move arms and legs; diagnosed with C6‐level incomplete paraplegia (ASIA B)	24 h postoperatively	HDS, further decompression in second surgery	Post‐surgery improvement noted; strength improved to 3/5 in right U/Ext, 4/5 in left U/Ext, 2/5 in right leg, and 3/5 in left leg by day 3 (ASIA C)

Jun et al. [8]	2020	49‐F Hypertension	Cervical disc herniation at C6‐C7 level with spinal cord compression	ACDF at C6‐C7	Not specified	Ankle clonus + 4, knee jerk + 4, sensory deterioration Bilateral L/Ext motor Grade 0 (ASIA A)	Immediate postoperative	HDS + emergency laminoplasty	Full recovery of motor power and sensory deficit (ASIA E)

Liao et al. [9]	2020	51‐M	OPLL C2–C4 Cervical spinal stenosis	C3‐C4 posterior laminectomy decompression	—	Left hemiplegia (ASIA D)	Postoperative	Methylprednisolone Mannitol neurotrophic drug	Stable neurological function Muscle strength normal (ASIA E)

Kalidindi and Sath [10]	2020	63‐M Well‐controlled diabetes	Cervical canal stenosis with OPLL Cord atrophy with myelomalacia changes	Decompression by laminectomy and fusion	Complete loss of MEP Immediately after laminectomy	Complete tetraplegia (ASIA A)	Immediate postoperative	IV methylprednisolone	Stable vital sign No improvement Neurological function (ASIA A)

Sepulveda et al. [11]	2020	12 m‐M with ventriculoperitoneal shunt and encephalopathy	Arachnoid cyst causing spinal cord compression	Posterior cervical decompression and arachnoid cyst fenestration	Not specified	Right arm monoplegia (ASIA D)	Immediate after surgery	Oral dexamethasone and ketorolac After diagnosis, IV dexamethasone	Improvement in right arm mobility Chronic neuralgic pain in right arm (ASIA E)

Busack and Eagleton [12]	2020	63‐M Smoking and poorly controlled hypertension	Severe cervical stenosis from C2‐C3 to C5‐C6	C3–C6 laminectomy and C2‐T1 posterior Fusion	MEPs and SSEPs were monitored throughout the procedure	Lack of Sensation and 0.5 motor strength of all muscle group and 4.5 of bilateral deltoids (ASIA C)	90 min after incision	Intravenous and oral dexamethasone	2.5 strength of bilateral lower extremities, upper extremities return to 5.5 strength (ASIA E)

Acharya et al. [13]	2021	49‐M Progressive myelopathy for 8 years	OPLL	C3–C7 laminectomy	Not specified	Acute quadriparesis postop, spasticity (ASIA C)	Postoperative Day 3	HDS	Regained preop Neurological baseline within 7 days (ASIA D)

Singh et al. [14]	2022	59‐F Hypertension Type 2 diabetes mellitus Parathyroidectomy	OLF causing cervical spinal stenosis C3–C6	Posterior decompressive laminectomy	Not specified	Severe hypotension Bradycardia Tetraparesis Paralysis (ASIA C)	Postoperative Day 2	Dexamethasone MAP above 100	Severe tetraparesis Grade 4 strength In right leg (ASIA C)

Singh et al. [14]	2022	66‐F Hypertension	OPLL with spinal cord compression C3–C5	C6‐C7 Anterior C4 corpectomy	Not specified	Decreased strength in left hand Bladder retention (ASIA C)	Postoperative Day 2	Dexamethasone MAP above 100	Partial recovery, able to ambulate with a walker (ASIA D)

So et al. [2]	2022	61‐M	Signal change and significant spinal cord compression at C3–5; large ruptured disc at C6‐7 with severe cord compression	ACDF at C4‐5 and C6‐7	No	Near complete quadriplegia (ASIA A)	Immediate postoperative	Additional posterior decompression of the cervical spine, total laminectomy at C4–6, subtotal laminectomy at C7, LMSF at C4–6, intravenous methylprednisolone	Full recovery in 2 months (ASIA E)

Dahapute et al. [15]	2022	63‐M	OPLL C6 fracture	PCDF from C2‐T2	MEP status changed during laminectomy	Quadriplegia Sensory level at C5 (ASIA A)	Immediate after surgery	Dexamethasone, ICU	No improvement in motor status (ASIA A)

Goyal et al. [16]	2022	39‐F Diabetes	Cervical myelopathy due to prolapsed intravertebral disc at C5‐C6	ACDF	Not specified	Worsening of power in all four limbs post‐surgery (ASIA C)	Immediate postoperative	Dexamethasone Baclofen and steroids	Power improved 4/5 in all limbs (ASIA D)

Lei et al. [17]	2022	54‐M	CSM due to herniation of C4–C7 disc	ACCF C4–C7	Not specified	Inability to move arms and legs Muscle strength 3/5 in upper and lower limb (ASIA C)	7 days post operation	HDS mannitol mecobalamin + PCDF 11 days after initial surgery	Muscle strength improved to 4/5 in limbs and 5/5 in legs after second surgery (ASIA D)

Hsin et al. [1]	2023	61‐M	Severe left C6‐C7 neural exit canal stenosis	ACDF C6/C7	Yes, no alert	Numbness, hemisensory loss, radiculopathy (ASIA D)	Postoperative Day 6	Pregabalin	Not specified

Tanaka et al. [3]	2023	81‐M	Severe spinal canal stenosis due to OPLL at C3–C5	C3–6 double‐door laminoplasty with C5 laminectomy	Yes, intraoperative MEP showed > 70% reduction in bilateral abductor pollicis brevis, > 95% in abductor hallucis	Strong quadriplegia (ASIA A)	Immediate postoperative	Steroid administration	Six months later, the Brunnstrom stage was two for the upper right and lower left limbs and three for the upper left limb (ASIA C)

Kumar et al. [18]	2023	48‐F Obesity Grade 2	Multilevel cervical cord compression	Posterior cervical decompression	Not specified	Quadriplegia (ASIA A)	Immediate Postoperative	HDS and monitoring MAP above 85	Able to walk Weakness Paresthesia in upper limbs (ASIA D)

Guerrero and Prasad [19]	2024	60‐F Hypertension Well‐controlled asthma Type 2 diabetes Transient ischemic attack	Disc bulges at C5‐C6 and C6‐C7 with myelomalacia and multilevel cervical foraminal narrowing	ACDF C5–C7	Not specified	Progressive loss of fine motor control Quadriplegia Urinary retention (ASIA C)	After surgery	IV dexamethasone therapy for two weeks Physiotherapy	Significant improvement in balance and quadriplegia (ASIA D)

*Note:* IOM: intraoperative monitoring, U/Ext.: upper extremity, L/Ext.: lower extremity, and m: month.

Abbreviations: ACCF, anterior cervical corpectomy and fusion; ACDF, anterior cervical discectomy and fusion; HCD, herniated cervical disc; HDS, high‐dose steroid; LMSF, lateral mass screw fixation; OPLL, ossification of posterior longitudinal ligament.

## 2. Case Presentation

**Figure 1:**
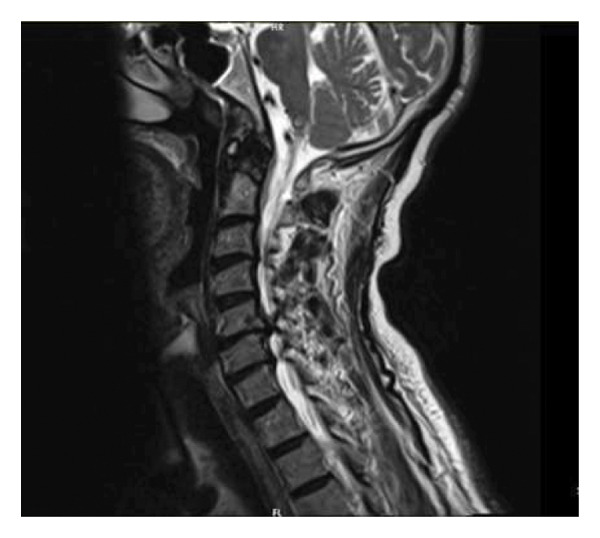
Preoperative T2‐W MRI: severe canal stenosis is seen at C5/C6 level and a disc bulge is causing mild canal stenosis and moderate bilateral neural foramen stenosis at C6/C7 level.

**Figure 2:**
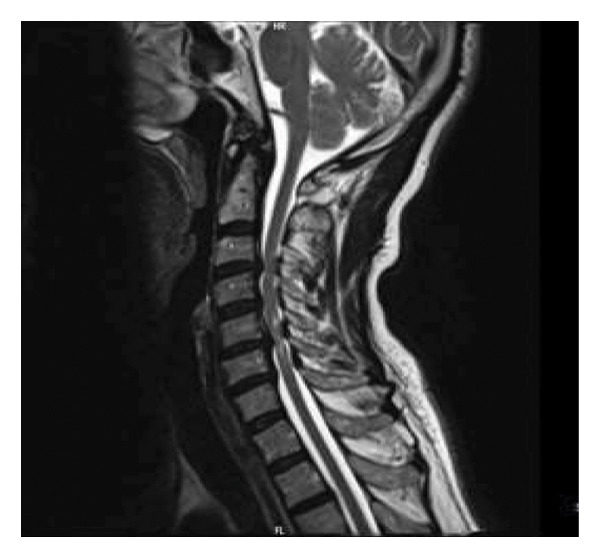
Preoperative T2‐W MRI: disc bulging is seen at C3/C4 and C5/C6 level and signal changes at C5/C6 level are seen in favor of myelopathy.

**Figure 3:**
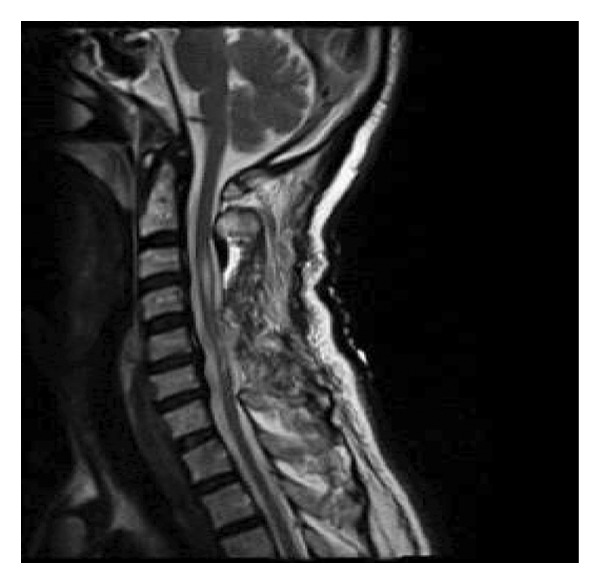
Postoperative T2‐W MRI: hyperintense signal change is seen at C3/C4 level.

A 61‐year‐old Iranian male presented with a four‐month history of progressive neck pain accompanied by gradually worsening left‐sided upper and lower limb weakness and paresthesia. Importantly, there was no history of recent acute trauma or an inciting event associated with the onset or progression of these neurological symptoms. His past medical history was significant for a remote, stable cervical trauma (an isolated C5 spinous process fracture sustained 12 years prior, managed conservatively without neurologic sequelae), peptic ulcer disease, chronic nonsteroidal anti‐inflammatory drug (NSAID) use, long‐term opioid consumption for chronic pain management, and a significant 30 pack‐year smoking history. Neurological examination on admission revealed symmetric motor strength of 4/5 in all extremities, left‐sided paresthesia without a defined sensory level, intact sacral sensation and proprioception, brisk deep tendon reflexes, bilateral positive Hoffmann’s signs, and sustained ankle clonus, consistent with American Spinal Injury Association (ASIA) Impairment Scale Grade D. Cervical MRI demonstrated hypertrophy of the ligamentum flavum and asymmetric disc bulging at the C3‐C4 level, resulting in moderate canal stenosis. Severe canal stenosis and foraminal narrowing were observed at the C5‐C6 level, accompanied by atrophic changes and T2 hyperintensity of the spinal cord, consistent with myelopathy (Figures 1 and 2). Approximately 18 h after admission, the patient underwent C3–C6 total laminectomy and C7 subtotal laminectomy under general anesthesia (propofol 120 mg, fentanyl 150 mg, and atracurium 30 mg) without concomitant fusion due to advanced age and relative spinal stability. Immediately postoperatively, upon emergence from anesthesia, a new neurological deficit was noted, consisting of left hemiplegia with left upper limb plegia (0/5) and left lower limb paresis (2/5), while motor and sensory functions on the right side and sacral sensation (S4‐S5) remained completely intact. This corresponded to a decline to ASIA Impairment Scale Grade C. An emergent postoperative MRI confirmed adequate decompression and new linear hyperintense signals in the cervical cord, particularly at C3‐C4, consistent with WCS (Figure 3). High‐dose intravenous methylprednisolone was initiated postoperatively immediately after the MRI, following a protocol of a 2‐gram loading dose followed by 500 mg every 6 h for 48 h. Gradual neurological improvement was observed during hospitalization, with motor strength improving to 2/5 in the left upper limb and 3/5 in the left lower limb while the right side remained at 5/5 and sacral sensation was preserved. This neurological status was consistent with an improvement back to ASIA Impairment Scale Grade D. The patient was discharged with a referral for rehabilitation, although follow‐up data were unavailable.

## 3. Discussion

WCS represents an uncommon but devastating complication following anterior or posterior cervical decompression. It is defined by acute postoperative neurological decline without identifiable intraoperative injury [5]. In 2013, Chin et al. first described this syndrome in a patient who developed quadriplegia after anterior cervical discectomy and fusion at C4–C6. The authors suggested that ischemia–reperfusion injury was responsible for the new T2 hyperintense signals observed on cervical MRI [5, 7].

The pathophysiology of WCS is not yet fully elucidated; however, the most widely accepted explanation attributes it to ischemia–reperfusion injury that occurs following decompressive surgery. The sudden restoration of blood flow to chronically compressed spinal cord tissue may cause microvascular damage and oxidative stress, resulting in secondary neuronal injury. Prolonged compression leads to ischemia and metabolic dysfunction within the spinal cord, and the subsequent reperfusion introduces reactive oxygen species (ROS) that exacerbate oxidative damage. Lipid peroxidation of neuronal membranes, together with the release of inflammatory mediators, further contributes to cellular injury and neurological decline. Following decompression, levels of proinflammatory cytokines such as tumor necrosis factor‐α (TNF‐α) and interleukin‐1β (IL‐1β) increase significantly, as demonstrated in experimental models of chronic spinal cord compression. The severity of WCS appears to be closely related to the duration of ischemia, the volume of affected tissue, and the metabolic demands of the spinal cord at the time of reperfusion [6, 20]. Further experimental evidence supports this mechanism. A 2015 rat model demonstrated that sudden decompression after prolonged ischemia caused extensive gray matter necrosis and neurological deterioration, reinforcing the hypothesis that reperfusion injury plays a central role in WCS pathogenesis [8].

Recent evidence suggests that ischemia–reperfusion alone may not fully explain the spectrum of WCS. Chronic compression induces microvascular rarefaction, blood–spinal cord barrier (BSCB) disruption, axonal demyelination, and glial activation, all of which reduce the tolerance of spinal cord tissue to sudden reperfusion, and preoperative T2 hyperintensity (myelomalacia) has been repeatedly identified as a strong predictor of postoperative neurological deterioration, likely reflecting irreversible structural vulnerability of the cord prior to surgery. Furthermore, abrupt decompression may cause rapid mechanical expansion of the edematous cord, stretching small penetrating vessels and provoking a secondary inflammatory response, while mitochondrial dysfunction and calcium influx during reperfusion have been proposed as major contributors to neuronal apoptosis. Collectively, these data support a multifactorial pathogenesis involving chronic ischemia, microvascular injury, loss of autoregulation, abrupt reperfusion, oxidative stress, inflammation, and apoptosis [19, 21].

Although risk factors are not yet fully defined, advanced age, chronic hypertension, and diabetes mellitus have been proposed as potential contributors to the development of WCS [20]. In the twenty cases we presented, 13 patients were male, 6 had hypertension, and 4 had type II diabetes mellitus (Table 1). The mean age was 56.6 years, with only one pediatric case (12 years old). These findings suggest that WCS predominantly affects middle‐aged and elderly patients, with a higher prevalence in males. The presence of vascular and metabolic comorbidities such as hypertension and diabetes may increase the spinal cord’s susceptibility to ischemia–reperfusion injury. In elderly individuals, chronic oxidative stress resulting from the gradual deterioration of homeostatic mechanisms may increase the risk of developing WCS [20].

Recent analyses have also highlighted several surgery‐related and radiologic factors associated with an increased risk of WCS. Among these, preoperative T2 hyperintensity or myelomalacia remains the strongest radiographic predictor, as it reflects reduced vascular reserve and chronic structural vulnerability of the spinal cord. Severe or multilevel stenosis—particularly in cases of ossification of the posterior longitudinal ligament (OPLL) or congenital canal narrowing—further contributes to long‐standing ischemia and impaired autoregulation. A prolonged duration of symptoms exceeding 12 months has similarly been linked to irreversible microstructural injury. Additionally, posterior decompression procedures appear overrepresented in reported WCS cases, likely due to the abrupt dorsal shift and expansion of the cord after laminectomy. The intraoperative loss of neuromonitoring signals, even in the absence of mechanical trauma, may also indicate a transient ischemic event that predisposes patients to postoperative deterioration. Collectively, these findings suggest that WCS most frequently occurs in individuals with longstanding compression, compromised microvascular physiology, and radiologic evidence of chronic spinal cord injury [21, 22].

Using a compilation of reported cases including those summarized in Table 1, several practical strategies have been proposed to minimize the risk of WCS in patients undergoing cervical decompression surgery. Preoperatively, careful radiological assessment should focus on T2 hyperintensity (myelomalacia), the degree and chronicity of cord compression, and the presence of ossification or congenital canal narrowing, with high‐risk patients specifically counseled about WCS [19]. Optimization of systemic and vascular comorbidities, including blood pressure and glycemic control, may further reduce susceptibility to ischemia–reperfusion injury. Intraoperatively, gradual decompression is recommended when anatomically feasible to avoid abrupt reperfusion, and multimodal intraoperative neurophysiological monitoring (INOM)—somatosensory evoked potentials and motor evoked potentials (SSEPs and MEPs)—should be employed to detect early ischemic changes, with any significant signal loss prompting immediate reassessment and correction. Maintaining adequate mean arterial pressure (MAP) (≥ 85 mmHg) and considering neuroprotective anesthetic strategies, such as propofol infusion, may help reduce BSCB permeability and oxidative injury. Postoperatively, immediate MRI at the first sign of neurological deterioration is essential to exclude compressive lesions and assess cord edema, while early high‐dose corticosteroid therapy combined with hemodynamic support may improve recovery outcomes. Importantly, in selected cases with persistent deficits following anterior decompression, timely secondary posterior decompression has been associated with superior neurological recovery compared with corticosteroid therapy alone, highlighting the value of early recognition and appropriate surgical intervention [8, 23].

In reviewing the reported cases of WCS, a clear pattern emerges when comparing neurological outcomes between patients who underwent secondary posterior decompression and those managed with corticosteroid therapy alone. Among patients who developed WCS following anterior cervical decompression, secondary posterior decompression was consistently associated with superior neurological recovery, with most showing full or near‐complete improvement. In contrast, outcomes were markedly poorer among patients treated solely with high‐dose corticosteroids, of whom only a minority achieved full recovery. This contrast suggests that, when WCS develops after anterior decompression, the addition of posterior decompression may mitigate residual mechanical stress on the cord and improve perfusion dynamics more effectively than pharmacologic management alone. The distinction in outcomes between these two groups underscores the importance of considering timely secondary decompression, particularly in patients with severe preoperative stenosis or persistent postoperative cord signal changes.

From a surgical standpoint, several technical considerations may help minimize the risk of WCS during cervical decompression. Gradual decompression is recommended, where the posterior laminae or anterior disc material is removed incrementally rather than in a single step, allowing the chronically compressed cord to accommodate restored perfusion gradually. Gentle handling of the spinal cord and minimizing excessive traction or manipulation of the dura are crucial, as abrupt expansion of edematous or myelomalacic tissue can precipitate reperfusion injury. In multilevel decompressions, sequencing the levels from least to most compressed has been suggested to reduce sudden hemodynamic shifts within the cord. Intraoperative neuromonitoring (SSEPs and MEPs) should guide these maneuvers, with any significant change prompting immediate adjustment of decompression strategy or temporary suspension to allow spinal cord recovery [9, 21].

To minimize the risk of developing WCS, several preoperative and intraoperative strategies can be implemented. One promising approach is remote ischemic preconditioning (RIPC), which has demonstrated neuroprotective effects against spinal cord ischemia. In a prospective randomized controlled trial involving patients undergoing decompressive spinal surgery, RIPC was applied through three cycles of five‐minute upper limb ischemia followed by five minutes of reperfusion. The study revealed significantly lower serum levels of neuron‐specific enolase and S‐100B in the RIPC group, indicating reduced neuronal injury and improved spinal cord tolerance to ischemia [6].

Another key preventive measure involves maintaining optimal MAP during and after decompression surgery. Similar to the management of cerebral hyperperfusion syndrome, maintaining hemodynamic stability is essential to preserve spinal cord perfusion and prevent secondary ischemic damage. Although definitive evidence on the ideal MAP threshold in spinal cord injury is limited, several studies recommend sustaining MAP above 85 mmHg to ensure adequate perfusion pressure [1].

According to Busack and Eagleton, patients with spinal cord injury exhibit a higher autoregulatory threshold for perfusion compared to healthy individuals, whose normal autoregulatory range lies between 70 and 150 mmHg [12]. Similarly, Hsin et al. reported that maintaining MAP at or above 85 mmHg improved neurological recovery and facilitated the restoration of intraoperative neuromonitoring signals [1].

During spinal surgery, INOM serves as a valuable tool for real‐time assessment of spinal cord integrity. Monitoring SSEPs and MEPs allows early detection of possible spinal cord compromise such as direct trauma, excessive manipulation, or ischemia. A significant loss or alteration of these signals during surgery may signal impending neurological injury, warranting immediate intraoperative intervention [6].

The differential diagnosis of WCS includes several potential conditions that can present with similar postoperative neurological deficits. These encompass iatrogenic spinal cord injury resulting in cerebrospinal fluid leakage or pseudomeningocele formation, cerebrovascular accidents, and previously unrecognized demyelinating disorders. However, the primary differential considerations for WCS are iatrogenic injury and postoperative spinal cord compression caused by an epidural hematoma. Advanced imaging studies, particularly MRI, play a crucial role in excluding these possibilities, helping to distinguish WCS from other causes such as epidural hematoma or cerebrovascular insult [4, 10, 14].

The diagnosis of WCS is primarily based on the appearance of hyperintense lesions on T2‐weighted MRI following decompressive spinal surgery. Although WCS is rare, certain diagnostic features have been proposed to guide identification. These include the presence of significant preoperative spinal cord compression, rapid onset of postoperative paralysis—typically within a few hours of decompression—and concurrent motor and sensory deficits involving both upper and lower limbs. Other possible causes of neurological deterioration must be excluded before establishing the diagnosis. A characteristic feature supporting the diagnosis is the observation of neurological improvement, either partial or complete, following timely administration of high‐dose methylprednisolone combined with dehydration and neurotrophic therapy [6].

Given its potential to cause severe and sometimes irreversible neurological deficits, increasing clinical awareness of WCS is essential. Once paralysis occurs, prompt recognition and early therapeutic intervention play a critical role in optimizing recovery outcomes. High‐dose corticosteroid therapy remains the cornerstone of initial management, while the need for additional surgical intervention depends on the patient’s response to medical treatment. Furthermore, it is strongly recommended that surgeons inform patients about the possibility of WCS as part of the preoperative consent process for decompressive spinal procedures [7].

Based on our analysis of the twenty cases presented in this study (Table 1), four patients required a secondary surgical intervention in addition to corticosteroid therapy. Among these, three had initially undergone anterior cervical decompression and subsequently received posterior decompression as a secondary procedure. Two of these patients achieved complete neurological recovery, while the third demonstrated significant improvement. The remaining patient, who underwent both primary and secondary posterior decompression, also showed satisfactory progress.

In comparison, among the sixteen patients who did not undergo a second surgery, only one achieved full recovery with corticosteroid therapy alone. These observations suggest that in patients who develop WCS following anterior cervical decompression, secondary posterior decompression may lead to superior neurological outcomes compared with medical management alone.

Based on accumulated case reports, reviews, and expert opinions, several practical strategies have been proposed to further minimize the risk of WCS in patients undergoing spinal decompression surgery. Preoperatively, detailed radiological assessment should focus on T2 hyperintensity (myelomalacia), the degree and chronicity of cord compression, and the presence of ossification or congenital canal narrowing, with high‐risk patients specifically counseled about WCS. Optimization of systemic and vascular comorbidities, including blood pressure and glycemic control, may reduce susceptibility to ischemia–reperfusion injury. Intraoperatively, gradual decompression is preferred when feasible to avoid abrupt reperfusion, and multimodal INOM—including SSEPs and MEPs—should be employed to detect early ischemic changes, with any significant signal loss prompting immediate reassessment and correction. Maintaining adequate MAP (≥ 85 mmHg) and considering neuroprotective anesthetic strategies, such as propofol infusion, may help reduce BSCB permeability and oxidative injury. Postoperatively, immediate MRI should be performed at the first sign of neurological deterioration to exclude compressive lesions and assess cord edema, and early high‐dose corticosteroid therapy combined with hemodynamic support may improve recovery outcomes. In selected cases with persistent deficits after anterior decompression, early secondary posterior decompression may lead to superior neurological outcomes compared with corticosteroid therapy alone [23, 24].

A limitation of this case report is the lack of long‐term follow‐up data, which restricts the ability to evaluate the sustained neurological recovery and functional outcomes of the patient. Although gradual improvement in motor strength was observed during hospitalization, the absence of post‐discharge follow‐up precludes assessment of the durability of recovery, potential late complications, or long‐term rehabilitation outcomes. This limitation highlights the need for future studies and case reports to include structured follow‐up to better understand the prognosis of patients developing WCS after cervical decompression.

## Ethics Statement

This manuscript does not include personal or medical details about any identifiable individual. The patient provided consent for the writing and publication of this article.

## Consent

Apart from age and gender, no identifying details were included in the manuscript. The patient provided written consent for the utilization of their hospital records and imaging as per the journal’s patient consent guidelines.

## Conflicts of Interest

The authors declare no conflicts of interest.

## Funding

This research was not funded by any particular grants from public, commercial, or not‐for‐profit organizations.

## Data Availability

The data that support the findings of this study are available on request from the corresponding author. The data are not publicly available due to privacy or ethical restrictions.

## References

[1] Hsin L. Y. , Samynathan C V. V. , and Yilun H. , White Cord Syndrome: A Treatment Dilemma, Cureus. (2023) 15, no. 4, 10.7759/cureus.38177. PMC10224717 37252488

[2] So J.-S. , Kim Y.-J. , and Chung J. , White Cord Syndrome: A Reperfusion Injury Following Spinal Decompression Surgery, Korean Journal of Neurotrauma. (2022) 18, no. 2, 10.13004/kjnt.2022.18.e36. PMC9634306 36381466

[3] Tanaka S. , Yoshida S. , Tomio R. , Mukasa A. , and Nishimatsu T. , White Cord Syndrome After Cervical Laminoplasty in an 81-Year-Old Man, Cureus. (2023) 15, no. 6, 10.7759/cureus.40386. PMC10344683 37456440

[4] Vinodh V. P. , Rajapathy S. , Sellamuthu P. , and Kandasamy R. , White Cord Syndrome: A Devastating Complication of Spinal Decompression Surgery, Surgical Neurology International. (2018) 9, no. 1, 10.4103/sni.sni_96_18, 2-s2.0-85060727385. PMC6057171 30090668

[5] Chin K. R. , Seale J. , and Cumming V. , ‘White Cord Syndrome’ of Acute Tetraplegia After Anterior Cervical Decompression and Fusion for Chronic Spinal Cord Compression: A Case Report, Case Reports in Orthopedics. (2013) 2013, no. 1, 10.1155/2013/697918. PMC3603640 23533882

[6] Giammalva G. , Maugeri R. , Graziano F. et al., White Cord Syndrome After Non-Contiguous Double-Level Anterior Cervical Decompression and Fusion (ACDF): A “No Reflow Phenomenon”?, Interdisciplinary Neurosurgery. (2017) 7, 47–49, 10.1016/j.inat.2016.12.001, 2-s2.0-85006963329.

[7] Papaioannou I. , Repantis T. , Baikousis A. , and Korovessis P. , Late-Onset “White Cord Syndrome” in an Elderly Patient After Posterior Cervical Decompression and Fusion: A Case Report, Spinal Cord Series and Cases. (2019) 5, no. 1, 10.1038/s41394-019-0174-z, 2-s2.0-85068853340. PMC6461846 31240122

[8] Jun D. S. , Baik J.-M. , and Lee S.-K. , A Case Report: White Cord Syndrome Following Anterior Cervical Discectomy and Fusion: Importance of Prompt Diagnosis and Treatment, BMC Musculoskeletal Disorders. (2020) 21, 1–5, 10.1186/s12891-020-3162-3. PMC7066844 32164644

[9] Liao Y.-X. , He S.-S. , and He Z.-M. , ‘White Cord Syndrome’, A Rare But Disastrous Complication of Transient Paralysis After Posterior Cervical Decompression for Severe Cervical Spondylotic Myelopathy and Spinal Stenosis: A Case Report, Experimental and Therapeutic Medicine. (2020) 20, no. 5, 10.3892/etm.2020.9218. PMC7507019 32973939

[10] Kalidindi K. and Sath S. , ‘White Cord Syndrome’ of Acute Tetraplegia After Posterior Cervical Decompression and Resulting Hypoxic Brain Injury, Asian Journal of Neurosurgery. (2020) 15, no. 03, 756–758, 10.4103/ajns.ajns_240_20. 33145248 PMC7591215

[11] Sepulveda F. , Carballo L. , Carnevale M. , and Yañez P. , White Cord Syndrome in a Pediatric Patient: A Case Report and Review, Radiology Case Reports. (2020) 15, no. 11, 2343–2347, 10.1016/j.radcr.2020.08.047. 32994838 PMC7501484

[12] Busack C. D. and Eagleton B. E. , White Cord Syndrome Causing Transient Tetraplegia After Posterior Decompression and Fusion, Ochsner Journal. (2020) 20, no. 3, 334–338, 10.31486/toj.19.0081. 33071672 PMC7529124

[13] Acharya S. , Kaucha D. , Sandhu A. S. , Adsul N. , Chahal R. S. , and Kalra K. L. , Misdiagnosis of “White Cord Syndrome” Following Posterior Cervical Surgery for Ossification of the Posterior Longitudinal Ligament: A Case Report, Surgical Neurology International. (2021) 12, 10.25259/sni_268_2021. PMC8247735 34221575

[14] Singh R. D. , Arts M. P. , and de Ruiter G. C. , Delayed-Onset White Cord Syndrome After Anterior and Posterior Cervical Decompression Surgery for Symptomatic Ossification of Spinal Ligaments: Illustrative Cases, Journal of Neurosurgery: Case Lessons. (2021) 1, no. 19, 10.3171/case2113. PMC9245768 35854839

[15] Dahapute A. A. , Balasubramanian S. G. , and Annis P. , White Cord Syndrome Following Posterior Decompression and Fusion for Severe OPLL and an Acute Traumatic Cervical Injury–A Case Report and Review of Literature, Surgical Neurology International. (2022) 13, 10.25259/sni_692_2022. PMC9699864 36447894

[16] Goyal N. , Chaturvedi J. , Kandwal P. , Gupta P. , Kaushal A. , and Kumar M. , ‘White Cord Syndrome’: A Rare Catastrophic Complication Following Anterior Cervical Discectomy and Fusion, Neurology India. (2022) 70, no. Suppl 2, S306–S309, 10.4103/0028-3886.360940. 36412386

[17] Lei C.-Z. , Gong D.-J. , and Zhou Y.-F. , Late-Onset White Cord Syndrome Following Anterior Cervical Discectomy and Fusion: A Case Report, Experimental and Therapeutic Medicine. (2022) 25, no. 1, 10.3892/etm.2022.11770. PMC9798147 36605533

[18] Kumar V. , Rai A. , and Dhatt S. S. , White Cord Syndrome—An Unforeseen Complication and Diagnosis of Exclusion: A Case Report and Review of Management, Egyptian Journal of Neurosurgery. (2023) 38, no. 1, 10.1186/s41984-023-00234-9.

[19] Guerrero A. M. C. and Prasad V. , White Cord Syndrome as a Rare Complication Post Cervical Spinal Decompression Surgery: A Case Report, Cureus. (2024) 16, no. 9, 10.7759/cureus.70304. PMC11437701 39345806

[20] Epstein N. E. , Reperfusion Injury (RPI)/White Cord Syndrome (WCS) Due to Cervical Spine Surgery: A Diagnosis of Exclusion, Surgical Neurology International. (2020) 11, 10.25259/sni_555_2020. PMC7568108 33093997

[21] Bagherzadeh S. , Rostami M. , Jafari M. , and Roohollahi F. , ‘White Cord Syndrome’ as Clinical Manifestation of the Spinal Cord Reperfusion Syndrome: A Systematic Review of Risk Factors, Treatments, and Outcome, European Spine Journal. (2025) 34, no. 1, 50–63, 10.1007/s00586-024-08461-w. 39266775

[22] Maldonado-Pérez A. , Campos J. , Murray G. , Estronza S. , and Pastrana E. A. , White Cord Syndrome: Myelopathy Caused by an Ossified Posterior Longitudinal Ligament After Posterior Cervical Laminectomy and Fusion, Cureus. (2025) 17, no. 3, 10.7759/cureus.80295. PMC11978165 40201890

[23] Gerardi R. M. , Giammalva G. R. , Basile L. et al., White Cord Syndrome After Cervical or Thoracic Spinal Cord Decompression. Hemodynamic Complication or Mechanical Damage? An Underestimated Nosographic Entity, World Neurosurgery. (2022) 164, 243–250, 10.1016/j.wneu.2022.05.012. 35589039

[24] Jain M. , Tripathy S. K. , Varghese P. , Naik S. , Sahoo D. R. , and Singh A. K. , White Cord Syndrome Following Long Posterior Decompression, Journal of Orthopaedic Case Reports. (2024) 14, no. 9, 14–18, 10.13107/jocr.2024.v14.i09.4712. PMC11381079 39253650

